# Application of a novel, continuous-feeding ultraviolet light emitting diode (UV-LED) system to disinfect domestic wastewater for discharge or agricultural reuse

**DOI:** 10.1016/j.watres.2019.01.006

**Published:** 2019-04-15

**Authors:** Thi Minh Hong Nguyen, Poonyanooch Suwan, Thammarat Koottatep, Sara E. Beck

**Affiliations:** a Environmental Engineering and Management Program, School of Environment, Resources and Development, Asian Institute of Technology, P.O. Box 4, Khlong Luang, Pathumthani, 12120, Thailand; b Department of Environmental Microbiology, Eawag: Swiss Federal Institute of Aquatic Science and Technology, 8600, Dubendorf, Switzerland

**Keywords:** UV-LEDs, Flow-through, Decentralized, Domestic wastewater, Water reuse, Fouling

## Abstract

In many low-income countries, the poor conditions of sanitation systems have been a significant cause of mortality since they accelerate waterborne disease transmission. Developing sanitation systems in these countries is a pressing concern in both the public and private sectors.

This research investigated a decentralized domestic wastewater treatment system using ultraviolet light-emitting diodes (UV-LEDs). Although UV-LED disinfection has become more widespread in recent years, it is a novel approach for domestic wastewater treatment. Domestic wastewater was pretreated by a low-cost pretreatment system with an inclined settler and a sand filter prior to feeding a novel flow-through UV LED reactor. At an inlet flow rate of 30 L/h, the COD, TSS, and turbidity of the effluent were 17.7 mg/L, 3.0 mg/L, and 3.9 NTU, respectively. UV transmittance at 285 nm was enhanced from 29.1% to 70.4%, improving the influent quality for UV LED disinfection.

The flow-through UV LED reactor was operated at various flow rates from 10 to 50 mL/min, resulting in applied UV doses of 69.4 to 47.8 mJ/cm^2^ respectively. These doses are sufficient for inactivating total coliforms in the wastewater to meet the water reuse guidelines for agriculture for both processed food crops and non-food crops.

Fouling, which was observed starting at 2 d of operation, decreased the disinfection efficacy to 27% after 25 days of continuous operation. Of the fouling layer, 67% was attributed to organic matter, in contrast to previous fouling studies with mercury UV lamps in which the fouling layer consisted primarily of inorganic compounds. The fouling was reversed by off-line citric acid cleaning for 4 h after every 400 h of continuous operation.

## Highlights

• Evaluated inclined settler and sand filter for pre-treating domestic wastewater.
• Disinfected domestic wastewater with a continuous feeding flow-through UV LED reactor.
• Investigated fouling characteristics of a continous feeding flow-through UV LED reactor.
• Confirmed that citric acid cleaning is a viable fouling reversal method to restore efficacy.

## Graphical abstract

**Figure:**
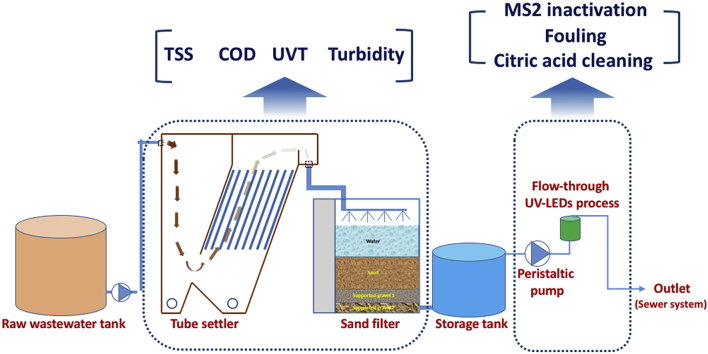

## 1 Introduction

In a recent study, more than 380 disease outbreaks related to waterborne parasitic protozoa were documented globally in a six-year period between 2011 and 2016 (Efstratiou et al., 2017). Additionally, 87% of total global mortality cases attributed to diarrhea came from South Asia and Sub-Saharan Africa (Troeger et al., 2017). There is no doubt that poor water supply and sanitation systems are a root cause leading to these public health issues (Yang et al., 2012). Therefore, developing sanitation systems, especially disinfection processes, plays a critical role in addressing the issue.

Ultraviolet (UV) disinfection is one of most common and most effective technologies for water and wastewater disinfection (Song et al., 2016). Although other conventional disinfection technologies have been widely used such as ozone, chlorine oxide, chlorine and peracetic acid; they are all chemical containing and/or by-product forming processes. UV disinfection is considered environmentally-friendly as a noncorrosive treatment option free of disinfectant residuals and disinfection byproducts (Liberti et al., 2003). For this reason, there have been case studies on the application of UV disinfection for wastewater reclamation. In Southern Italy, UV disinfection was successful in removing protozoan parasites for unrestricted reuse of municipal wastewater in agriculture with no formation of disinfection byproducts (Liberti et al., 2003). A case study of water reuse in the Middle East evaluated UV disinfection for inactivating enteric pathogens remaining after treatment by constructed wetlands (Azaizeh et al., 2013). UV disinfection also showed a high performance in disinfecting swine secondary effluent for water reuse under alkaline-controlled conditions in Catarina, Brazil (Bilotta et al., 2017).

These previous studies involved traditional UV lamps containing mercury vapor, which is extremely toxic to human health and the environment when it comes to lamp breakage during operation or discharging (Nance et al., 2012). The United Nations Environmental Programme (UNEP), through the Minamata Convention on Mercury, is working to control mercury waste and eliminate the release of mercury into air, water, and land by 2020 (United Nations Environment Programme, 2013). Although the Minamata Convention does not specifically address mercury UV lamps, it raises awareness of the harmful effects of mercury and encourages organizations and governments to adopt mercury-free products. Additionally, mercury UV lamps are highly fragile, temperature variation sensitive, and they contain a large footprint. Thus, UV light emitting diodes (UV-LEDs) have been recently considered as a potential novel UV source offering features that could overcome the shortcomings of these lamps, including high durability, robust designs, lower voltage requirements, and lack of mercury (Chevremont et al., 2013; Würtele et al., 2011). However, studies applying UV LEDs for wastewater disinfection processes in real conditions are limited; in particular, there is a lack of case studies involving flow-through UV-C LED processes.

Ultraviolet disinfection is not completely free from drawbacks. Wastewater constituents such as solid particles, humic acid and turbidity can reduce the inactivation efficacy of the UV disinfection process due to absorption, scattering and shielding effects (Carré et al., 2018; Mamane et al., 2006). Consequently, in applications of UV disinfection, proper and efficient up-stream treatment processes are often a decisive factor for success. Therefore, this research pretreated the domestic wastewater with a low-cost, conventional system consisting of an inclined tube settler and a slow sand filter prior to feeding the novel flow-through UV LED reactor.

Due to the high total settling area in comparison with other types of sedimentation, inclined settlers have been widely applied to enhance settling velocities of clarification processes especially without support from chemical flocculation. There have been a variety of inclinations used in inclined settlers in the literature ranging from 8° to 60°; however, most of industrial manufactures set their products at 60° inclination since it reduces the footprint of the processes and ensures sludge flow (Smith and Davis, 2013; Smith et al., 2013). Slow sand filtration has been considered an effective conventional technology in water and wastewater treatment for treating a wide range of physical, chemical and biological pollution (Haig et al., 2011; Li et al., 2018; Paredes et al., 2016). Moreover, slow sand filtration processes are simple for operation and maintenance, have low operating costs, and can be used as tertiary treatment in wastewater treatment plants. Sand grain sizes range from 0.1 to several millimeters generally (Haig et al., 2014).

Maintenance requirements are also an integral part of putting UV LED disinfection technology into practice. A very important consideration during operation of UV disinfection processes is fouling. The growth of foulant on quartz sleeves due to the increase of temperature during operation could significantly reduce the UV dose applied due to a decrease of UV transmittance of the sleeves (Nessim and Gehr, 2006). Although it has been hypothesized that LEDs will not be affected by fouling as much as mercury lamps because heat generated during operation is dissipated on the opposite side as the light source (Arik et al., 2004), to the best of our knowledge, no attempt has been made to evaluate fouling during application of a UV LED disinfection process in wastewater treatment. Thus, the objectives of this work were to (1) investigate the performance of a low-cost pretreatment system, (2) study the disinfection efficacy of a flow-through UV LED system in continuous operation, (3) determine the fouling period and fouling layer constituents and (4) propose an environmentally-friendly cleaning method for removing the fouling layers.

## 2 Materials and methods

### 2.1 Wastewater source

Domestic wastewater was collected from a manhole at the Asian Institute of Technology's (AIT) sewer system (Pathumthani Province, Thailand). The AIT campus, with a population of 2000–2,500, generates wastewater with a mixture of greywater and blackwater mainly from dormitories, offices and a cafeteria. Wastewater samples collected daily were analyzed, giving the characteristics shown in Table 1. All parameters were determined (n = 18) following standard methods for the examination of water and wastewater (APHA, 2012).

**Table 1:** Characteristics of influent wastewater, n = 18.

Parameters	pH	Temp (^o^C)	Turbidity (NTU)	TSS (mg/L)	COD (mg/L)	UV Transmittance at 285 nm (%)	Total coliform (MPN/100 mL)	*Escherichia coli* (MPN/100 mL)	MS2 coliphage (PFU/mL)
Analysis method	4500B—H^+^	–	2130B	2540D	5220C	Spectrophotometry	9221C	9221F	USEPA1602
Average ± SD	7.3 ±0.3	29.0 ±4.2	63.3 ±16.6	82.7 ±25.0	141.8 ±53.5	25.2 ±9.4	1.6 × 10^5^ ±0.7 × 10^5^	9.6 × 10^4^ ±5.5 × 10^4^	21.8 ±8.9
Range	7.2 – 7.51	26.5 – 27.8	36.0 – 98.1	44.0–136.3	96 – 272	11.6 – 43.7	8.6 × 10^4^ - 2.8 × 10^5^	3.0 × 10^4^ - 1.8 × 10^5^	12–34

### 2.2 Pre-treatment system with inclined tube settler and slow sand filtration

**Fig. 1:**
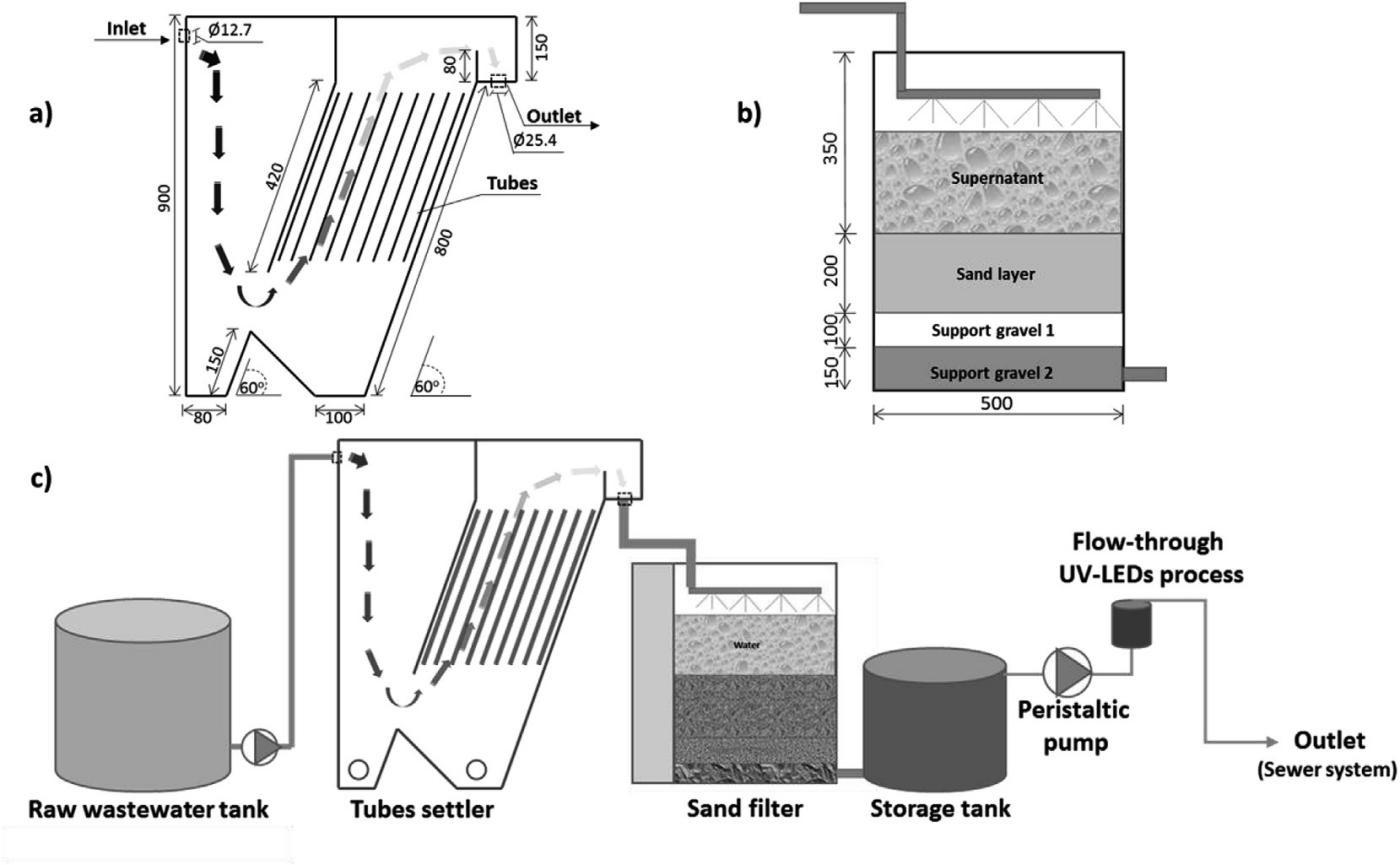
Designed scheme of a) tube settler b) sand filter c) overall experimental setup. Units given in mm.

Prior to entering the UV-LED unit, the raw wastewater was first stored in a raw wastewater tank and then pumped with a peristaltic pump through a conventional system with an inclined tube settler and a slow sand filter (Fig. 1). The gravitational tube settler consisted of 117 tubes (400 mm length x 12.7 mm diameter) inclined at 60° and fed from the bottom. The respective total working volume and settling surface were calculated at 0.076 m^3^ and 1.87 m^2^. The tube settler acted as a sedimentation unit to remove extensive suspended solids, which would have disturbed the UV-LED disinfection process. The tube settler overflow was gravity-fed to the sand filter. The sand filter was constructed in three layers including a sand layer with sand size, D_10_ – D_60_, of approximately 0.4–0.8 mm at a thickness of 0.2 m, a small supported gravel layer with gravel size, D_10_ – D_60_, of 5–10 mm at a thickness of 0.1 m, and a large supported gravel layer with gravel size, D_10_ – D_60_, of 10–20 mm at a thickness of 0.15 m, in that order. D refers to the effective diameter; D_10_ and D_60_ mean 10% and 60% of the particles were finer than that size, respectively. The total surface area of sand filtration was 0.2 m^2^. Before pumping to the flow-through UV LED process, an appropriate amount of pretreated wastewater was stored in a storage tank. Since the UV LED process had lower capacity compared to the pretreatment system, the extra volume of pretreated wastewater was directly discharged into the sewer system. Schematics of the pretreatment units and the treatment system are given in Fig. 1.

The pretreatment system was first operated at three inlet flow rates of 30, 60, and 90 L per hour (L/h) to determine an optimal flow rate, which would generate an acceptable influent for the UV-LED unit. At these flow rates, the corresponding overflow rates of the tube settler were 0.016, 0.032 and 0.048 m^3^/m^2^.h respectively and the filtration rates of the sand filter were 0.15, 0.3 and 0.45 m^3^/m^2^.h, respectively. The pretreatment efficiency of the system was based on four parameters: total suspended solids (TSS), chemical oxygen demand (COD), turbidity and UV transmittance (UVT). The UVT (%) was calculated by Eq. (1). All parameters were determined (n = 6) following standard methods for the examination of water and wastewater (APHA, 2012)

(1) UVT _λ_ = 100 × 10^−a(λ)^

where UVT is the UV transmittance at wavelength **λ** and **a (λ)** is the absorbance of wastewater at wavelength **λ**, measured by a Hitachi U-2900 spectrophotometer (Tokyo, Japan).

Carré et al. (2018) demonstrated that the microorganism inactivation efficacy of a UV disinfection process was significantly enhanced when the turbidity was under 5 NTU while Mariel et al. (2012) indicated that the increase of turbidity in wastewaters from 9.9 to 16.2 NTU resulted in a 60% reduction in the disinfection performance of the UV process. Thus, the pretreated wastewater quality was designed to be less than 5 NTU in turbidity.

### 2.3 Flow-through UV-LED reactor

**Fig. 2:**
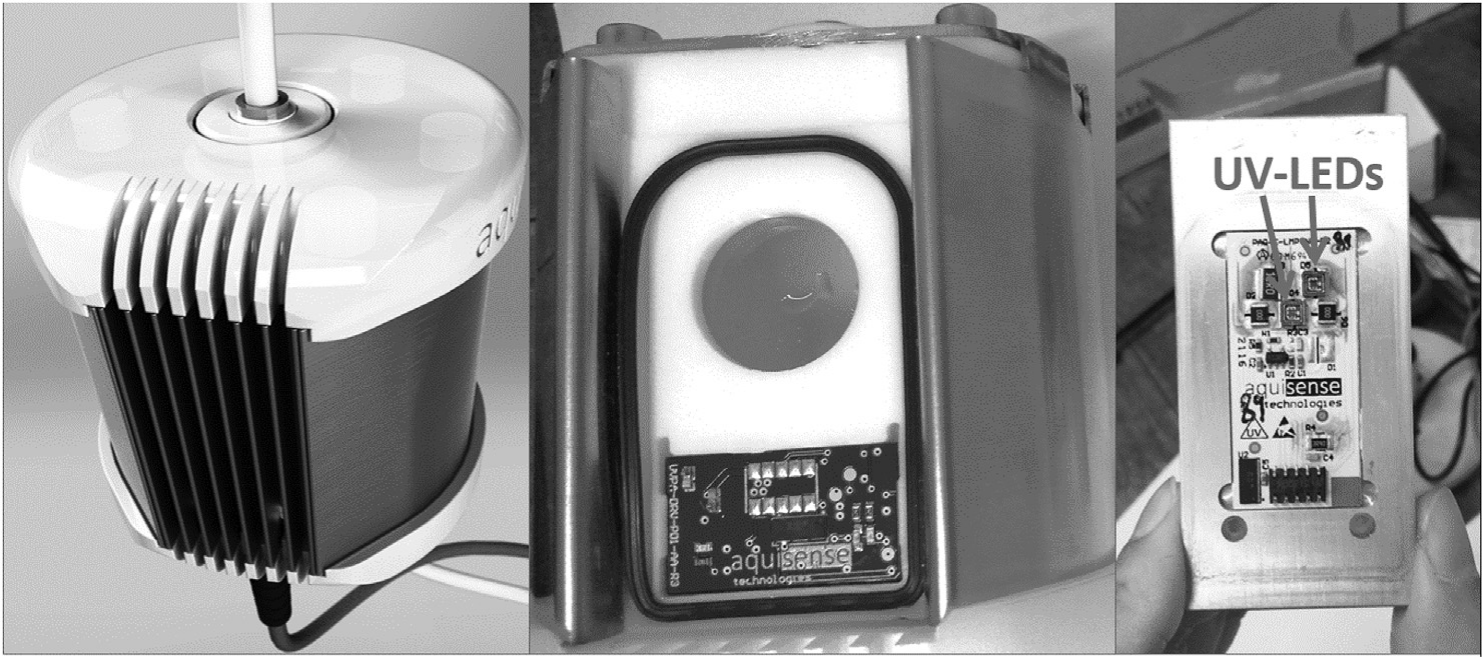
Flow-through UV LED reactor (AquiSense, USA).

**Fig. 3:**
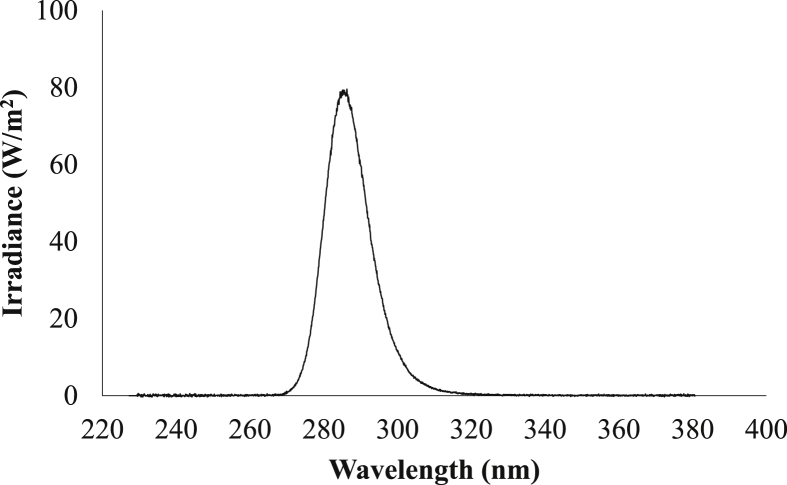
Spectral emission of UV-LEDs in the flow-through reactor.

The flow-through UV-LED reactor used in this study, shown in Fig. 2, was a commercially-available PearlAqua™ 6D (AquiSense Technologies, Kentucky, USA). The reactor contained two LEDs with the spectral emission given by the manufacturer shown in Fig. 3; they exhibited a peak wavelength emission at 285 nm (Fig. 3), a weighted-average spectral emission of 287 nm, and a full width at half-maximum (FWHM) of 13.5 nm. The LEDs have a rated effective lifetime of 10,000 h as given by the reactor manufacturer. The Pearl Aqua unit has an input voltage of 12V DC and a total optical output of 65 mW. The reactor contains a working volume of 68.6 mL, an inlet and outlet diameter of ¼ inch, and an outer length and diameter of 107 × 87 mm.

### 2.4 UV fluence determination

To evaluate the efficacy of the UV LED disinfection process, the UV doses applied by the flow-through reactor to the water samples were determined using a method called biodosimetry described previously (Oguma et al., 2016; USEPA, 2006). In this method, the flow-through reactor was challenged with a microorganism at specific flow rates and its log reduction from UV exposure was determined for each flow rate. In parallel, quasi-collimated beam experiments were conducted with a batch reactor to determine the dose-response of the microorganism in each water sample. The dose-response curve was then used as a standard curve to back-calculate the UV dose, or the reduction equivalent fluence (REF), delivered by the flow-through reactor at each flow rate.

Pure cultured male specific 2 (MS2) coliphage (ATCC 15597-B1, American Type Culture Collection, Virginia, U.S.) was used as the representative microorganism and biodosimeter for the disinfection studies. Male specific and F-specific coliphage have commonly been considered indicators of the presence of pathogens in addition to other popular microorganisms such as *E.coli*, fecal coliforms or Enterococci (Wu et al., 2011). MS2 coliphage also has much in common with mammalian viral pathogens, including enteroviruses, such as size, shape, isoelectric point, genome length, and morphology (Michen and Graule, 2010; Park et al., 2011). Moreover, MS2 is generally more resistant to environmental factors compared to other virus surrogates (Feng et al., 2003; Silverman et al., 2013).

The flow-through UV-LED reactor was operated at five different flow rates of 10, 20, 30, 40, and 50 mL/min. The treated wastewater was spiked with MS2 at a concentration of 10^5^ to 10^8^ PFU/mL. Corresponding UV doses applied by the flow-through process at each flow rate were estimated by correlating the log reduction of MS2 to a UV dose-response curve developed with the batch reactor experiments described below.

**Fig. 4:**
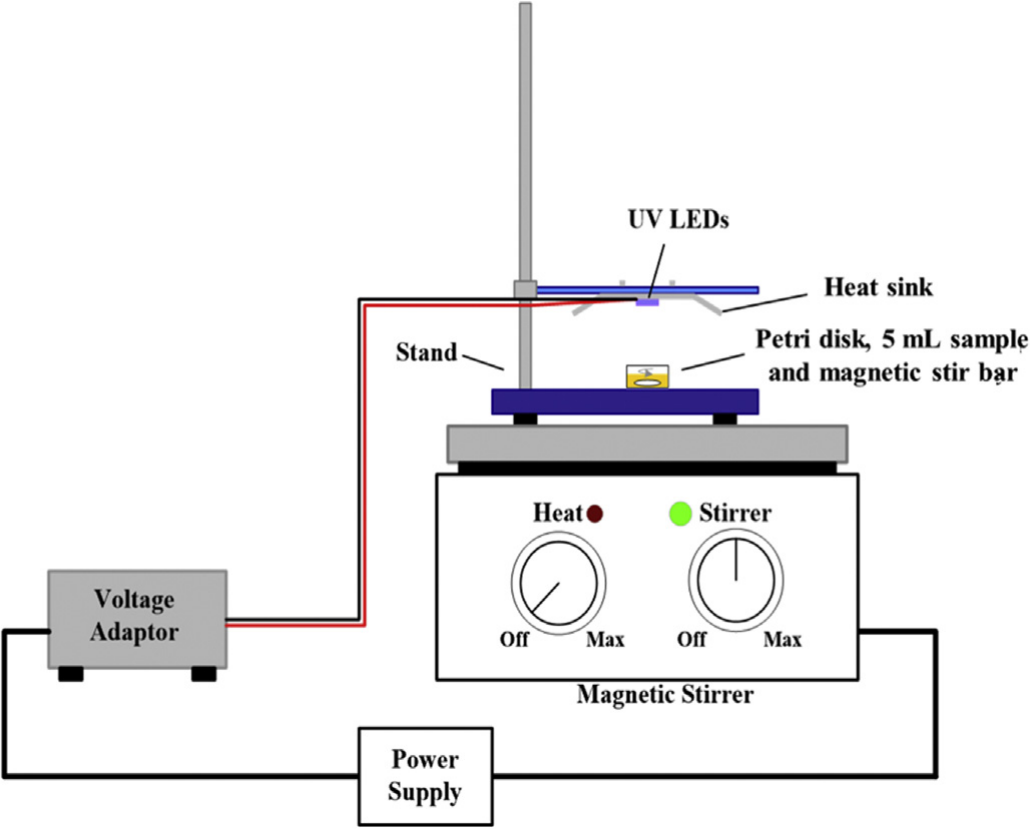
UV-LED lab-scale batch reactor.

The batch reactor illustrated in Fig. 4 contained a UV-LED unit purchased from TDS Light Company Limited (TDS-UV280-J9, Jiangsu, China) with nine LEDs set at a distance of 3 cm from the water sample surface. The spectral emission of the UV-LED batch reactor (Fig. S1) was measured at the National Institute of Metrology Thailand (NIMT) with a diode array spectroradiometer (CAS140CT–154), a cosine receptor probe (EOP-146) and a non-optical density filter (Instrument Systems, Germany). The LEDs in the batch reactor had a peak wavelength emission of 277.3 nm, a weighted average wavelength of 276 nm, a FWHM of 10.7 nm, and the radiation profile shown in Fig. S2. Incident irradiance at the center of the water sample was 0.30 mW/cm^2^ and the uniformity of the batch reactor irradiance, shown in Fig. S3, yielded a Petri factor of 0.876 for the 3.5 cm dishes (inner diameter 3.35 cm), which were cornered in the same position for each experiment by two secure microscope slides. Current and voltage inputs were controlled with a voltage regulator to stabilize the irradiance output; the unit was operated with an input current and voltage of 60 mA and 17 VDC, respectively.

Given the challenges of operating the batch reactor unit without an in-house radiometer as well as challenges associated with polychromatic actinometry calculations, the irradiance of the batch reactor was back-calculated using biodosimetry with MS2. The inactivation rate constant reported in the literature for a UV-LED device with the same peak wavelength emission (277 nm) and FWHM (10 nm) was 0.052 cm^2^/mJ (Beck et al., 2017). Using that inactivation rate in these experiments, the average irradiance across the surface of the water sample back-calculated from biodosimetry was 0.208 mJ/cm^2^ ± 0.0133 (n = 3). This value was comparable to the average irradiance *across the surface* of the water sample calculated from off-site radiometry (0.213 mJ/cm^2^) for a polychromatic UV source, adjusted to account for the Petri factor, reflection factor, and divergence factor (Linden and Darby, 1997; USEPA, 2006). These values were then used together with the water factor to determine the average irradiance applied *throughout* each water sample (Beck et al., 2017). The calculation did not include a collimation factor, as proposed by Kheyrandish et al. (2018) which was published a full year after these experiments were conducted. The irradiance was weighted germicidally, by the DNA absorbance, to calculate the UV doses for each batch experiment as described previously (Linden and Darby, 1997; USEPA, 2006). Weighting by the MS2 action spectrum instead of DNA absorbance would have had a negligible effect since the action spectrum of MS2 closely follows that of DNA and RNA within the spectrum emitted by these LEDs (Beck et al., 2016). To account for the slight differences in UVLED wavelength emission from the batch (277 nm) and the flow through reactors (285 nm), average germicidal irradiance was also calculated for a hypothetical batch reactor with the same LEDs as those used in the flow-through reactor. The ratio of average germicidal UV irradiances led to a wavelength correction factor of 0.97. Average germicidal irradiance would be slightly lower from LEDs emitting at 285 nm than those emitting at 277 nm since their peak emission is further from the peak of RNA absorbance (Beck et al., 2016).

Each batch reactor experiment exposed a 5 mL sample volume (6 mm depth), continuously stirred by a magnetic stir bar to create homogenous water sample. The UV doses (in mJ/cm^2^) were calculated as the product of the average irradiance (E_avg_, mW/cm^2^) and exposure time (s), where E_avg_ accounted for the UV absorbance of the water across the UV LED spectrum.

The log reduction of MS2 coliphage was estimated following Chick-Watson model:

(2) $$\text{Log}\;\text{reduction}=\text{log}\left(\frac{C_{0}}{C}\right)$$

where C_0_ and C are the MS2 coliphage concentration (PFU/mL) before and after exposure to the UV LEDs, respectively. The applied UV doses with the corresponding log reduction of MS2 were used to plot the UV dose response curves from which to determine the dose applied by the flow-through UV LED reactor.

### 2.5 Preparation and enumeration of the microorganism

The MS2 coliphage stock was propagated and enumerated following the U.S. Environmental Protection Agency (EPA) method for double agar layer (USEPA, 2001). For propagation, MS2 and the log-phage *E. coli* host (ATCC#700891) were inoculated in a mixture of phosphate buffered saline (PBS) and tryptic soy broth with magnesium chloride and ampicillin and streptomycin antibiotics per manufacturer's instructions. The mixture then was incubated at 37 ± 1 °C for 16–24 h. To separate MS2 from the bacterial cells, the mixture was centrifuged for 25 min at 3000 g. The supernatant was filtered through sterile 0.2-μm filters and stored in the freezer at −18 ± 2 °C.

### 2.6 Continuous-feeding operation process for the fouling and cleaning study

The reduction of the applied UV dose due to fouling over the continuous-feeding process was investigated by examining the decrease of disinfection efficacy through daily sampling. In these experiments, the flow-through reactor was operated at 30 ml/min with distilled water and MS2 as the challenge microorganism. Distilled water was used to eliminate disturbances and create a homogenous condition for comparison.

Since the UV dose of 20 mJ/cm^2^ is commonly applied for filtered effluent in wastewater disinfection for 3-log removal of microbial pathogens (Gehr et al., 2003; Lazarova et al., 1999), our study applied higher doses than that for ensuring pathogen disinfection. Therefore, the flow-through UV LED reactor was continuously fed at 30 mL/min with pretreated wastewater until the disinfection efficacy or fouling coefficient decreased to around 0.27, at which point the UV dose applied reached below 20 mJ/cm^2^ and cleaning was required. Fouling layers on the inside wall of the UV LED unit were removed to investigate fouling constituents (n = 2) using the inductively coupled plasma method (3120B) at Intertek (Bangkok, Thailand). A 50% hydrochloric acid (HCl) solution was used to dissolve the fouling layers to analyze the calcium (Ca), magnesium (Mg), iron (Fe) and phosphorus (P) content since they are assumed to be the dominant fouling materials (Lin et al., 1999a; Qiang et al., 2013). The total volatile solids (TVS, APHA method 2540E) content of the fouling layers was also examined in order to estimate the contribution of organic materials in the fouling layers. These parameters and other parameters of the pretreated wastewater were analyzed following the standard methods for the examination of water and wastewater, including hardness (2340C), iron (3500B) and total phosphorus (4500D-P) (APHA, 2012).

Citric acid was used to clean the fouled reactor. During the cleaning process, the UV LEDs were turned off and the flow-through reactor was processed with a 15% citric acid solution (pH < 2) at a flow rate of 30 mL/min. At each cleaning test, 1 L of the citric solution was prepared and recirculated. One cleaning experiment was conducted at each fouling period for a total of four cleanings with corresponding cleaning times of 2, 3, 4 and 12 h. The aim of this experiment was to determine the required time to reverse the fouling and return the reactor to its original condition using citric acid cleaning.

### 2.7 Statistical analysis

When comparing differences in removal efficiencies for the pretreatment system, one-way ANOVA analysis was used with a p-value < 0.05. One-way ANOVA analysis (p-value < 0.05) was also applied to determine significant differences in disinfection efficacies of the flow-through UV LED reactor among various operational flow rates.

## 3 Results and discussion

### 3.1 Pretreatment system performance

**Fig. 5:**
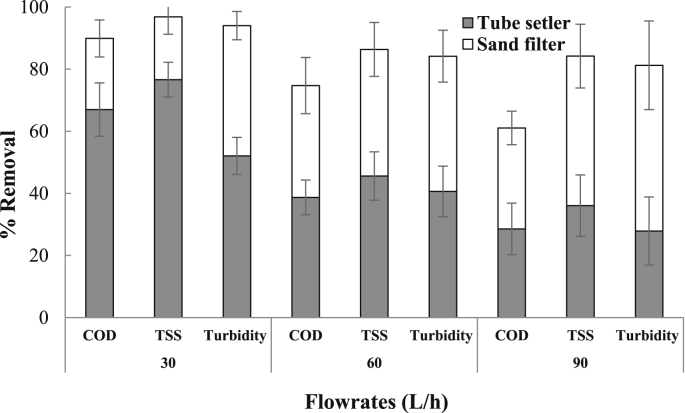
Overall treatment performance of the pretreatment system and contributions of tube settler and sand filter in COD, TSS and Turbidity removal (Average ± SD) at three inlet flow rates, n = 6.

The overall treatment performance of the tube settler and slow sand filter, in terms of TSS, COD and turbidity removal, is given in Table 2 and Fig. 5. The flow rate of 30 L/h had the highest removal efficiencies for TSS (96.8 ± 1.1%), COD (89.9 ± 3.4%), and turbidity (94 ± 2.3%). The other flow rates of 60 and 90 L/h led to lower removal efficacies of under 85% in most parameters. This indicates that the increase of inlet flow rate led to a decrease in treatment performance of the pretreatment system, as expected.

**Table 2:** Wastewater quality after each step of the pretreatment system, n = 6 (average ± SD).

Flow rate (L/h)	Pretreatment stage	SLR (m^3^/m^2^.h)^a^	Pretreated wastewater quality			
			COD (mg/L)	TSS (mg/L)	Turbidity (NTU)	UVT (%, 285 nm)
30	Raw wastewater	–	192.0 ± 70.1	101.2 ± 27.2	70.3 ± 17.0	29.1 ± 12.6
	Post tube settler	0.016	58.7 ± 9.7	23.2 ± 7.0	30.0 ± 5.0	39.5 ± 9.0
	Post sand filter	0.15	17.7 ± 3.2	3.0 ± 0.75	3.9 ± 0.7	70.4 ± 2.8
60	Raw wastewater	–	117.3 ± 8.3	68.2 ± 14.1	61.4 ± 19.0	25.1 ± 6.2
	Post tube settler	0.032	72.0 ± 8.8	37.7 ± 12.9	36.5 ± 14.0	30.8 ± 5.9
	Post sand filter	0.3	29.3 ± 6.5	9.0 ± 2.2	9.2 ± 1.2	47.7 ± 5.2
90	Raw wastewater	–	116.0 ± 14.1	78.7 ± 22.3	58.2 ± 14.9	21.5 ± 8.2
	Post tube settler	0.048	82.7 ± 12.0	50.2 ± 16.6	42.7 ± 13.3	26.7 ± 6.3
	Post sand filter	0.45	44.7 ± 12.9	12.0 ± 4.7	10.4 ± 0.7	44.9 ± 6.4

a SLR is the surface loading rate or overflow rate of tube settler and filtration rate of sand filter.

Since there was no support from up-stream coagulation-flocculation processes, the treatment output of the tube settler in this study was quite low in comparison with most of the literature. At an overflow rate of 0.016 m^3^/m^2^.h, the tube settler removed 76.6% of TSS (30.0 gTSS/m^2^.day), 67.0% of COD (51.3 gCOD/m^2^.day) and 52.1% of turbidity. Tarpagkou and Pantokratoras (2014) determined that a 60° inclined settler could increase the overall treatment efficiency compared to conventional designs, leading to an overall settling efficiency of 93%. The initial TSS concentration in that study was 1066 mg/L, which was 12 times higher than this study. Similarly, in another study, at an overflow rate of 0.2 m^3^/m^2^.h, 45° inclination, 1233 mg/L MLSS and a hydraulic retention time (HRT) of 20 min, 99.4% of TSS was removed (Fraji et al. (2013). In those cases, the application of chemical-based pretreatment was necessary due to the high TSS; however, in this study, coagulation and flocculation chemicals could have increased the turbidity in the effluent and impaired the light transmittance of UV-LEDs. The tube settler and sand filter in this study did not require any chemical-based support and the pretreatment efficacy was still efficient, resulting in effluent concentration of TSS, COD and turbidity of 3 mg/L, 17.7 mg/L and 3.9 NTU respectively at an operational flow rate of 30 L/h.

**Fig. 6:**
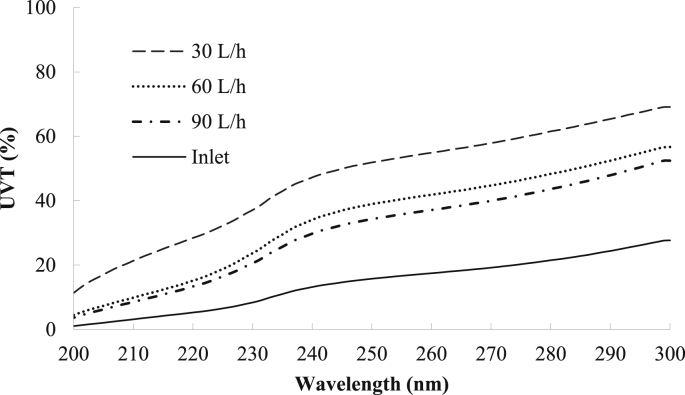
UV transmittance of treated wastewater at three inlet flow rates (Average), n = 12.

In analyzing the tube setter's and sand filter's contributions to overall removal efficiencies, the data revealed that the tube settler was more significant for removing TSS, turbidity and COD, whereas the sand filter was dominant in enhancing UV transmittance. For instance, at an operational flow rate of 30 L/h, the sand filter unit (0.15 m^3^/m^2^.h) contributed to the removal of 22.9 ± 6.0% of COD, 20.2 ± 5.6% of TSS and 42.0 ± 4.6% of turbidity from the raw wastewater; whereas the tube settler unit (0.016 m^3^/m^2^.h) contributed to the removal of over 66.9 ± 8.6% of COD, 76.5 ± 5.6% of TSS and 52.1 ± 6.0% of turbidity from the raw wastewater (Fig. 5). Regarding UV transmittance, a final UVT (at 285 nm) of 71.4% was obtained at a flow rate of 30 L/h (Fig. 6). The tube settler and sand filter accounted for a 10.5% and 30.9% UVT enhancement respectively, indicating that around 75% of the overall UVT enhancement of the pretreatment system was attributed to the sand filter. Rizzo et al. (2015) reported a better performance in which 62% of the UV absorbance (used to calculate UVT) was removed with sand filtration and graphene absorption at a 0.1 m/h filtration rate. In comparison to other pretreatment methods, subsurface wetlands treating household effluent reached a UVT (at 254 nm) of only 40% (Azaizeh et al., 2013, whereas a UVT (254 nm) of 57% was obtained when using sonication to treat trickling filter effluent for UV disinfection by Yong et al. (2008). This indicates that the conventional and low-cost pretreatment system in this study can be considered as a high-performance pretreatment option for UV LED disinfection.

The sand filtration effectiveness in lowering turbidity has been demonstrated in the literature. Li et al. (2018) reported turbidity of less than 1 NTU, which was significantly better than that of this study, from a filtration rate ranging from 0.5 to 0.2 m/h. However, Li et al. (2018) used a granular activated carbon (GAC) sandwich filter with 10 cm/30 cm/10 cm of sand/GAC/sand; the filtration media was more complex and thicker than in this study. In addition, Pompei et al. (2017) also reported 1 NTU turbidity of filtered water. The Pompei study used a thicker layer of sand (40 cm compared to 20 cm in this study) and the sand was a finer grain size of 0.21 mm compared to the 0.4–0.8 mm used in this study. Huisman and Wood (1974) concluded that the most effective sand size for slow sand filter ranging from 0.15 to 0.35 mm. These differences could explain why the Li and Pompei sand filtration systems reached lower effluent turbidities than in this study.

Given the above results, operating this pretreatment system with a flow rate of 30 L/h was optimal among the three flow rates tested. Therefore, this flow rate was selected for subsequent experiments. At 30 L/h, the pretreatment system could process 0.72 m^3^ of pretreated wastewater per day. This volume would account for at least 70% of average water consumption (and therefore generation) per household in Thailand, the study location (0.98 m^3^/day) (Metropolitan Waterworks Authority, 2017). The UV LED system had a significantly lower capacity compared to the pretreatment system; therefore, an appropriate amount of pretreated wastewater was stored in a storage tank for disinfection studies and the extra was directly discharged into the sewer system. The UV LED system could be scaled up to accommodate the total wastewater volume, however.

### 3.2 MS2 UV dose-response curve

**Fig. 7:**
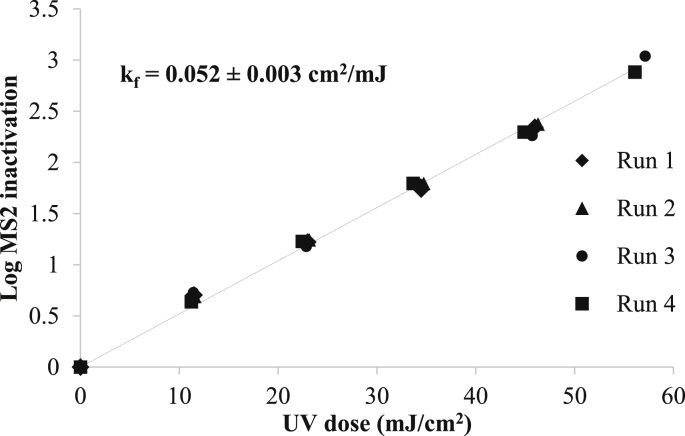
The dose-response curve developed from lab-scale batch reactor experiments with wastewater.

Through the lab-scale batch reactor experiments with wastewater, an MS2 UV dose response curve was developed, which demonstrated linear inactivation kinetics (Fig. 7). The fluence-based inactivation rate constant (k_f_) from the wastewater samples was exactly 0.052 ± 0.003 cm^2^/mJ (n = 4), which matched the designed inactivation rate constant from biodosimetry. In another study in which MS2 was inactivated with a high precision laser emitting monochromatic light at 280 nm, the inactivation rate constant, k_f_, was 0.043 cm^2.^/mJ; however, that source differed from these LEDs, which emit over a broader range down to 270 nm (Fig. 3), where the absorbance of RNA and therefore the inactivation of MS2 is higher (Beck et al., 2016). In another study with UV LEDs emitting at 275 nm, the MS2 inactivation rate constant was 0.035 cm^2^/mJ (Bowker et al., 2011; however, that study calculated the UV dose applied as if it were a monochromatic system whereas LEDs are polychromatic light sources offering broadband spectral emissions spanning across more than 20 nm.

### 3.3 Flow-through UV LED process disinfection efficiency

**Fig. 8:**
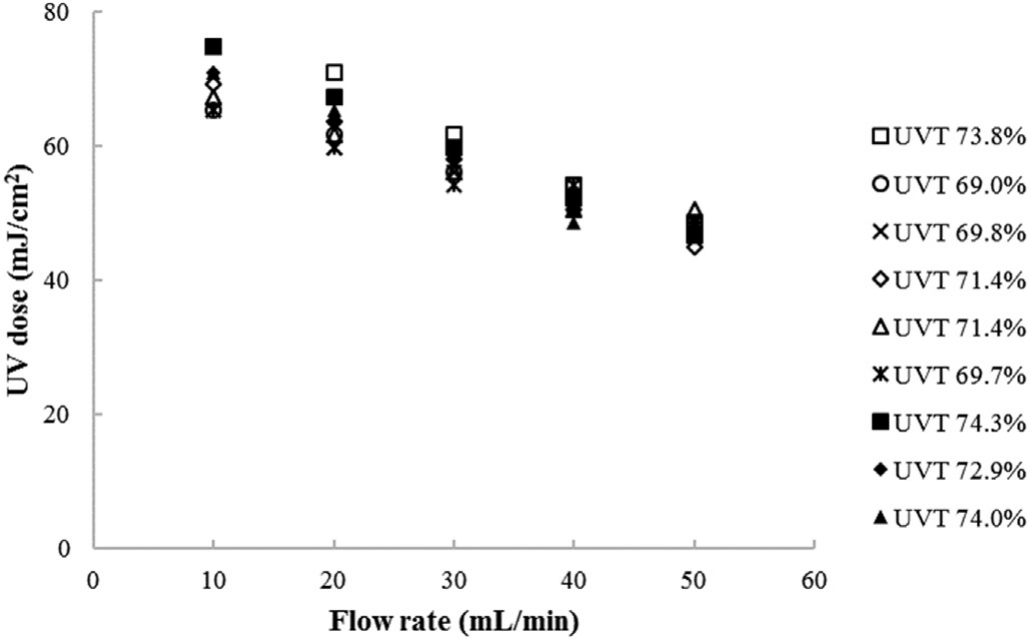
Inactivation performance of the flow-through UV LED process at different operational flow rates.

The disinfection performance of the flow-through UV LED reactor at the five different operating flow rates and their corresponding exposure time, log MS2 inactivation, and UV dose is given in Table 3 and Fig. 8.

**Table 3:** Disinfection performance of flow-through UV-LED process (n = 9).

Flow rate (mL/min)	Exposure time (s)	Log MS2 achieved(Log_10_ ± 1 SD)	UV dose(mJ/cm^2^)
10	412	3.7 ± 0.2	69.4 ± 3.8
20	206	3.4 ± 0.3	63.8 ± 3.7
30	137	3.1 ± 0.1	57.7 ± 2.4
40	103	2.8 ± 0.1	51.7 ± 2.1
50	82	2.6 ± 0.1	47.8 ± 1.6

As expected, the dose applied to the water sample decreased as the operating flow rates increased due to the lower UV exposure times. The lowest log MS2 inactivation was 2.6 ± 0.1-log at the highest flow rate tested of 50 ml/min, corresponding to a UV dose of 47.8 ± 1.6 mJ/cm^2^. From there, the MS2 log activation increased 1.45 times at a flow rate 5 times slower to 3.7 ± 0.2-log at a 10 ml/min, which was less efficient than the batch system. Although the UV dose increased with increasing exposure time, the dose response of the MS2 exhibited tailing. Lower inactivation efficiencies in flow-through reactors than in batch reactors have been reported previously for both low-pressure UV lamps and UV LEDs (Hijnen et al., 2006; Oguma et al., 2013). It is assumed that this is due to microbial aggregation, light shielding, and/or to heterogeneous hydraulic conditions within the reactor. Given that there was no mixing by a magnetic stirrer during operation of the flow-through process, microbial aggregations could occur especially when the operational time is increased. This finding was highly supported by Oguma et al. (2013) in a flow-through reactor and by Mattle and Kohn (2012) in a batch system. The inactivation efficacy of UV light could also be affected by light shielding, which prevents UV irradiation from reaching the target microorganism, particularly in waters with lower UV transmittance. Finally, heterogeneous hydraulic conditions could also play a role. The system could have been susceptible to short-circuiting, whereby a portion of the water exits the reactor before the average particle residence time. Short circuiting, and reduced disinfection efficacy, is more prominent at low flow rates (Iranpour et al., 1999). A UVLED system's configuration strongly affects its disinfection performance; specifically, the UV irradiance is stronger near the UV light source (Lee et al., 2018; Price et al., 2011). In this study, the maximum distance between the UV source and the reactor wall was 5.56 cm within a spherical reactor with a highly reflective material. UV reactors are typically designed to have a thin path length or a high UV transmittance. This study disinfected wastewater with a UVT (285 nm) of 70%, which was lower than the operational UVT recommended by the manufacturer of greater than 90%. However, tailing has also been seen in a cylindrical flow-through reactor with a maximum path length of 12 mm and UV LEDs of lower output (Oguma et al., 2013). Inactivation efficacies of UV LED systems vary with radiation profile, water characteristics, challenge microorganisms, system configurations, and sample volumes; future research in this area would be beneficial.

The concentrations of total coliforms and *E.coli* in the pretreated wastewater in this study both ranged from 0.8 × 10^4^ to 2.3 × 10^5^ MPN/100 mL, which is approximately 5 logs. Since the UV dose needed to inactivate *E.coli* is much lower than for MS2, at approximately 3.2 mJ/cm^2^ (at 280 nm) to 3.4 mJ/cm^2^ (at 260 nm) for 1-log inactivation (Beck et al., 2017; Oguma et al., 2013), the UV doses applied in this study of 69.4 mJ/cm^2^ before fouling or approximately 20 mJ/cm^2^ after fouling are sufficient for inactivating *E. coli* and total coliforms in pretreated wastewaters for agriculture use, which would meet the water reuse guideline for agriculture both for processed food crops and non-food crops of non-detected coliform in 100 mL samples (USEPA, 2012).

### 3.4 Fouling

**Fig. 9:**
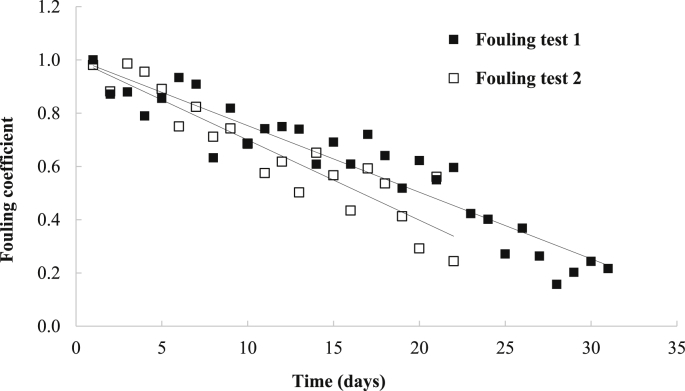
Coefficient of disinfection efficacies during continuous operation.

For fouling investigation tests, the flow-through UV LED process was continuously operated at a flow rate of 30 mL/min. During the continuous operation, disinfection efficacy of the process decreased from 3.7-log MS2 inactivation to approximately 1-log after 25 days, indicating fouling in the UV system; the fouling coefficient decreased from 1 to approximately 0.27 (Fig. 9). This reduction in disinfection efficacy was presumed due to fouling as opposed to LED aging because it was completely reversed as discussed in Section 3.5. The reduction in disinfection performance of the UV LED process was observed starting from the 2nd day of continuous operation (Fig. 9). The fouling appeared to follow first order kinetics, corresponding to the findings of Lin et al. (1999b). The estimated reduction of disinfection performance of the process was 0.12 ± 0.03 log MS2 inactivation per day (n = 52).

It is important to note that all previous fouling studies for ultraviolet disinfection processes were investigated with traditional UV lamps; therefore it is difficult to compare this study with others. Regarding the cause of fouling, Lin et al. (1999b) indicated that the increase of temperature during operation enhanced the precipitation of fouling materials. Consequently, the smooth surface of the quartz sleeve turned into a round surface and trapped water, while the high temperature enhanced the coprecipitation of foulant materials. In our study, the fouling layer appeared as a light brown amorphous film not only on the quartz plate, but also on the reactor wall. The manufacturer aimed to enhance inactivation efficacy with an interior surface of highly-reflective material; with a reflectivity of 95% at wavelengths of 250–300 nm, according to the manufacturer. Therefore, the fouling layer diminished the UV reflection off the reactor's wall and reduced the disinfection efficacy. Moreover, the presence of dominant fouling materials in pretreated wastewater (Table 4) and the system's hydrodynamic conditions including the low operational flow rate (30 mL/min) and bottom-feed operation contributed to the formation of the fouling layers in our study.

**Table 4:** Fouling materials in wastewater and fouling layer.

Parameters	Concentration in pretreated wastewater (n = 10)	Concentration from the fouling layer dissolved in HCl (n = 2) (Weight fraction of elements in the fouling layer, n = 2)
Calcium hardness	23.0 ± 2.3 (mg/L as CaCO_3_)	221.4 ± 47.4 mg/L (18.7 ± 3.1%, Ca)
Magnesium hardness	104.0 ± 8.7 (mg/L as CaCO_3_)	56.9 ± 19.7 mg/L (4.8 ± 1.9%, Mg)
Iron	0.22 ± 0.09 (mg/L)	1.44 ± 0.7 mg/L (0.12 ± 0.06%, Fe)
Total Phosphorus	1.53 ± 0.11 (mg/L)	4.13 ± 0.9 mg/L (0.35 ± 0.06%, P)
Total volatile solids	–	67 ± 14% (in total solids)

There have been many constituents in water that have caused fouling during UV disinfection processes, including calcium and magnesium hardness, iron, phosphorus and other elements and minerals in the matrix of the foulant whereas iron and hardness attributed to the most dominant fraction (Amin et al., 2010; Lin et al., 1999a; Qiang et al., 2013). Table 4 shows for this study the concentration of fouling elements in the pretreated wastewater, the concentration in the acid solution used to dissolve the fouling layer for analysis, as well as the fraction in the total weight of the fouling layer, including hardness, iron and phosphorus. This indicates that calcium compounds played an important role in the fouling layer composition (18.7%). Additionally, it has been reported that fouling layers dominantly consist of amorphous and inorganic films (Amin et al., 2010) whereas UV radiation is able to prevent the accumulation of organic foulants on quartz sleeves Lin et al. (1999b).

Other research studies have shown inorganic fouling materials to be the dominant constituents of the fouling layer; however, in this research volatile solids accounted for 67% of total solids in the fouling layers. Therefore, it is assumed that organic fouling materials could play a significant role. Given that UV irradiation could prevent organic fouling and the quartz plate was the closest part to light source, organic fouling on the quartz may have been insignificant. However, fouling occurred not only on the quartz plate but also on the interior surface of the reactor. Observations showed that the fouling layer was thin and rough on the quartz plate and a thicker uniform layer on the reactor's interior surface (Fig. S4). The thin but rough layer on the quartz plate was assumed to be due to precipitates. The thicker uniform layer on interior surface may have formed because of the reactor's design. The farthest point from LEDs to the interior surface was 5.56 cm; therefore, wastewater could have absorbed a majority of the UV irradiance before it reached the reactor's interior surface. As a result, organic fouling layers formed and increased over the long continuously operational period. Those layers could have trapped microorganisms following a similar mechanism to fouling on quartz sleeve in the study of Lin et al. (1999b). Consequently, fouling on the reactor's interior surface was considered an important cause of the reduction in disinfection performance over the continuous operation in this study. However, there remains a limitation in this study in that the fouling effects on the reactor's interior and the quartz cover could not be examined individually. Research into fouling layers developed in flow-through UV LED systems is limited. Therefore, more intensive studies on the fouling mechanisms of UV LED processes are necessary as UV LED disinfection technology is put into practice.

### 3.5 Proposed fouling reversal method

Citric acid has been utilized for metal ions chelating and as an alternative cleaning agent for calcium salts removal in a wide range of industrial activities, especially for food manufacturing (Vavrusova et al., 2017; Vavrusova and Skibsted, 2016). Citric acid is an organic compound, environmentally-friendly and biodegradable since it is derived from natural materials. Therefore, it is harmless to the environment. Other studies reported that citrate is an efficient calcium carbonate (CaCO_3_) scale inhibitor since it absorbs CaCO_3_ crystals and prevents the growth of crystal particles (Bassioni, 2010; Chaussemier et al., 2015; Wada et al., 2001). With regard to metal ions, Samsuri and Irawan (2004) determined that a solution of citric acid 4% (pH < 2, soaked for 5 h) could dissolve most iron complexes in montmorillonite whereas 0.1 M citric acid (soaked for 144 h) could dissolve 39% of total harbor sediment iron mineral in the study of Di Palma and Mecozzi (2007).

**Fig. 10:**
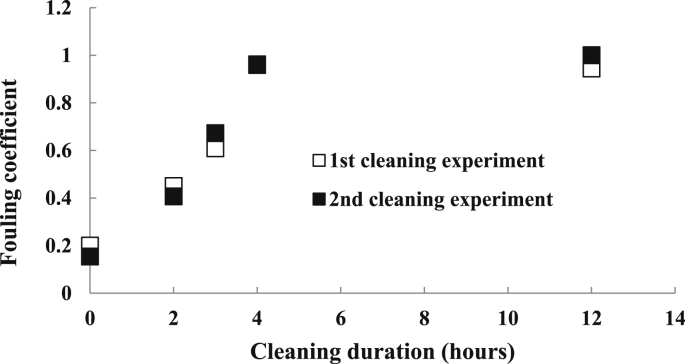
Inactivation efficacy of the flow-through UV LEDs process at four soaking periods (2, 3, 4, 12 h) (n = 2).

The UV LEDs were operated at a flow rate of 30 mL/min until the fouling effect reduced the applied UV dose to less than 20 mJ/cm^2^. Only 4 h of running with citric acid solution (15%, pH < 2) at a flow rate of 30 mL/min was required to recover the fouled UV LED reactor back to its original applied UV dose (69.4 mJ/cm^2^ at a flow rate of 10 mL/min with pretreated wastewater). A 12-h cleaning was also tested, indicating that there was no re-adsorption of the fouling constituents onto the reactor's wall and the quartz cover when the soaking period was greater than 4 h (Fig. 10). The U.S. EPA suggests that for continuous-feeding disinfection processes for traditional UV lamps, off-line cleaning with chemicals is required for up to 15 min; the cleaning frequency can vary depending on the water quality, the degree of fouling, and operating conditions (USEPA, 2006). For this study, cleaning the flow-through UV LED reactor for 4 h was required after 25 days of operation.

## 4 Conclusions

Through the above results, the following conclusions can be drawn:

(1) A conventional, low-cost system with an inclined settler and sand filter could be an appropriate option for pretreating domestic wastewater prior to UV LED disinfection. At a flow rate of 30 L/h, a 60^0^ inclined settler and a slow sand filter produced quality effluent for the subsequent UV LED disinfection. Specifically, TSS was decreased by 96.8 ± 1.1%, COD by 89.9 ± 3.4%, and turbidity by 94 ± 2.3%. As such, the effluent concentration of TSS, COD and turbidity were 3.0 ± 0.8 mg/L, 17.7 ± 3.2 mg/L and 3.9 ± 0.7 NTU, respectively. UVT at 285 nm was also significantly enhanced from approximately 29%–70%. The other inlet flow rates presented a lower treatment performance.
(2) In the flow-through UV LED process, 3.7-log MS2 inactivation was achieved at a flow rate of 10 mL/min, corresponding to an applied UV dose of 69.4 mJ/cm^2^. The increase of the inlet flow rate to 20, 30, 40, and 50 mL/min reduced disinfection performance to 3.4, 3.1, 2.8, and 2.6-log MS2 inactivation, respectively. The corresponding UV doses reached are sufficient for disinfecting water for agricultural use, which would meet the USEPA guideline for water reuse. Notably, with the same wastewater source, MS2 inactivation performance in the flow-through process did not follow the same trend compared to the batch reactor when it came to exposure time.
(3) It has been claimed that fouling in mercury lamp UV disinfection reactors is due primarily to inorganic constituents, taking place mostly on the quartz sleeve and resulting from the effects of temperature. In contrast, the fouling in this UV LED system was primarily organic, taking place both on the quartz and on the reactor's interior surface; it was also attributed in part to hydrodynamic conditions and the reactor's configuration. In this study, volatile solids content accounted for 67% of the total fouling constituents. Fouling of the flow-through reactor occurred within 2 days of operation with an average reduction of 0.12 ± 0.03 log MS2 inactivation per day. After 25 d continuous operation, the UV LED system fouling coefficient reduced from 1.0 to 0.27.
(4) Fouling reversal was possible with 4 h of citric acid soaking, restoring the UV LED flow-through reactor to its original disinfection efficacy. The required frequency of offline chemical cleaning, which depends on the water quality, the degree of fouling, and the applied dose, depends on the reactor configuration and UV emission source as well. This study can act as a starting point from which to base future research and future guidelines.

## Declaration of interests

The authors declare that they have no known competing financial interests or personal relationships that could have appeared to influence the work reported in this paper.

## Appendix A Supplementary data

WR46550 Manuscript.docx[37-38] WR46550 Manuscript.docx[37-38]
