# Supplementary material for: Application of a novel, continuous-feeding ultraviolet light emitting diode (UV-LED) system to disinfect domestic wastewater for discharge or agricultural reuse
Source: Water Res. 2019 Apr 15;153:53–62. doi: 10.1016/j.watres.2019.01.006 (PMC6382465; doi:10.1016/j.watres.2019.01.006)
Supplement: WR46550 Manuscript.docx[37-38] [file mmc1.docx]

**Supporting Information**

**S.1. Materials and Methods**

**S.1.1 UV fluence determination**

**Figure S1. Spectral emission of UV LEDs used in the lab-scale batch reactor as measured at the National Institute of Metrology, Thailand.**

**
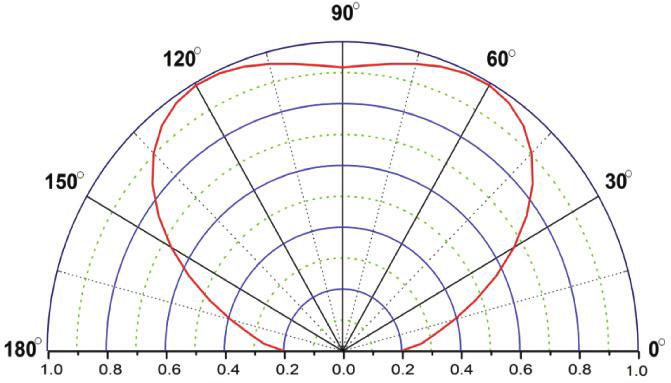
**

**Figure S2. Radiation profile of the UV LEDs used in the batch reactor as given by the manufacturer, TDS Lighting CO., LTD.**

**Figure S3. Irradiance surface profile of UVC LED radiation for the batch reactor.**

| **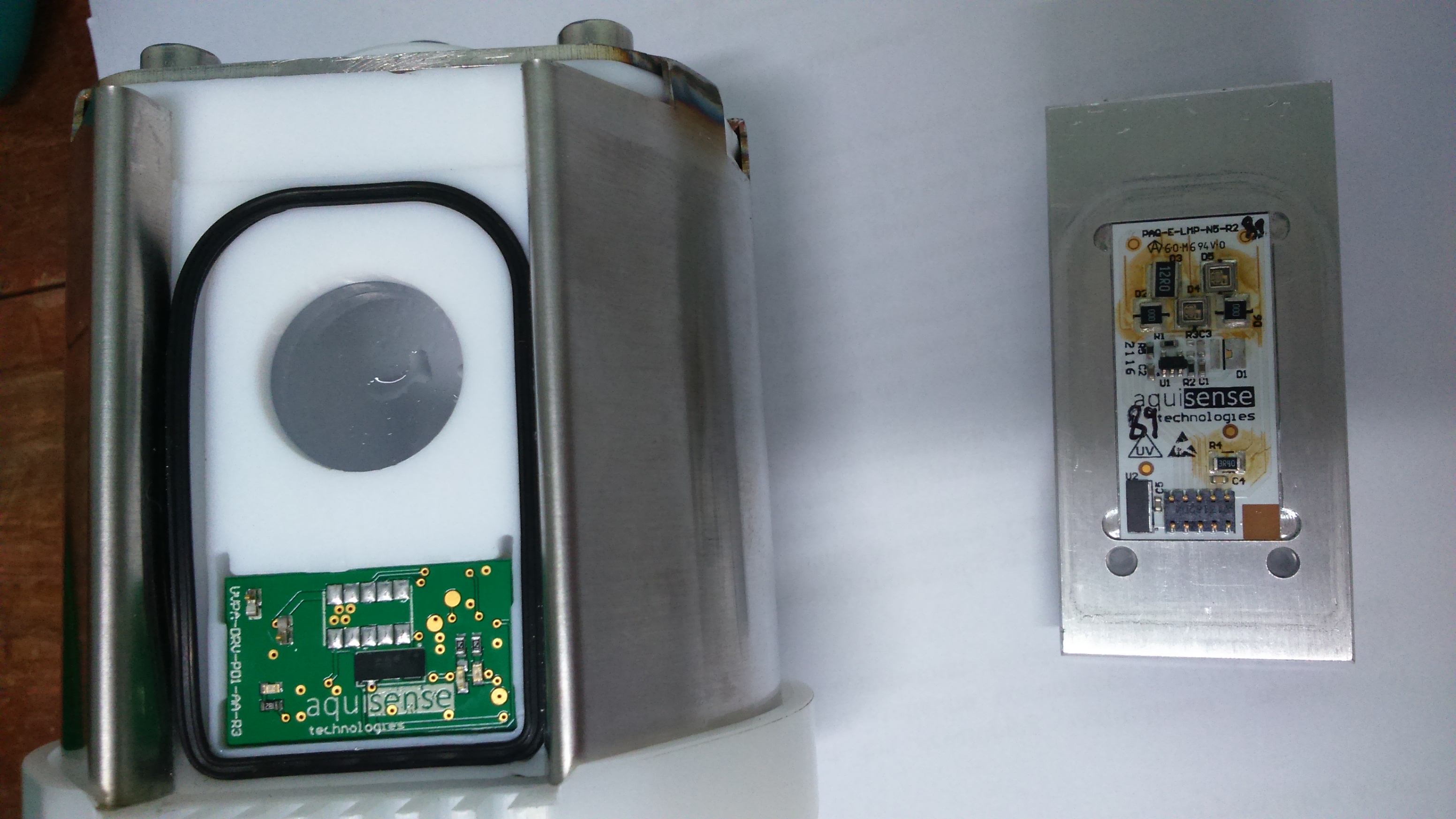** | **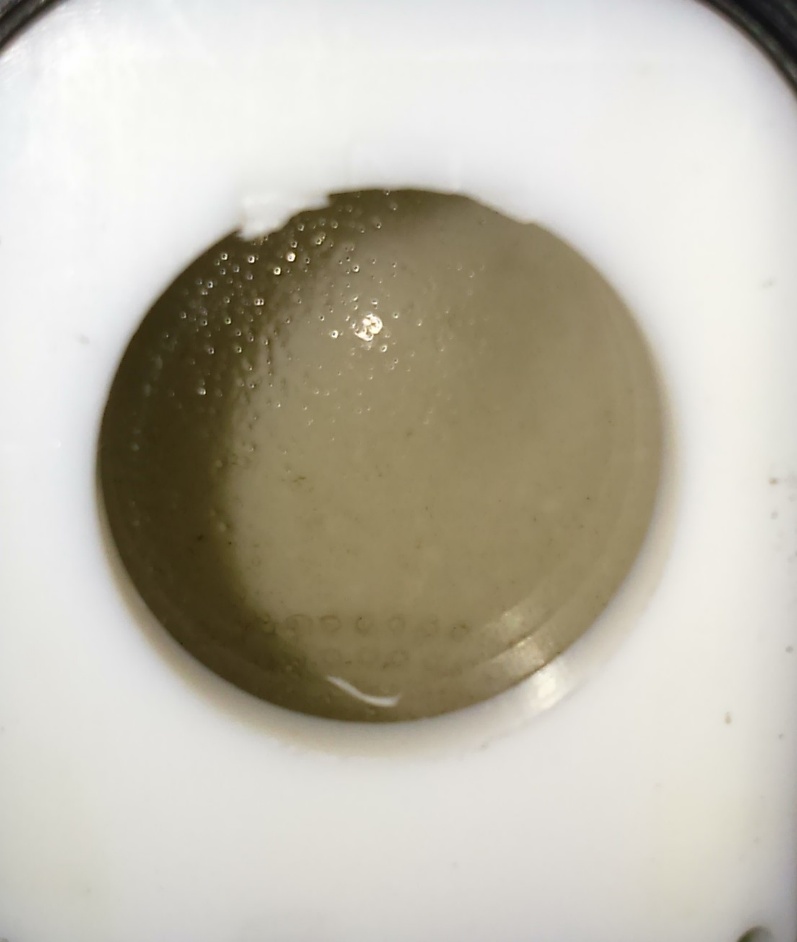** |
| --- | --- |

**Figure S4. Observaion of the UV LED reactor (left) in non-fouled (after cleaning) condition
(right) in fouled condition**
